# Turning cell cycle entry on its head

**DOI:** 10.7554/eLife.03475

**Published:** 2014-07-01

**Authors:** Cosetta Bertoli, Robertus Antonius Maria de Bruin

**Affiliations:** 1 **Cosetta Bertoli** is at the MRC Laboratory for Molecular Cell Biology, University College London, London, United Kingdom; 2 **Robertus Antonius Maria de Bruin** is at the MRC Laboratory for Molecular Cell Biology, University College London, London, United Kingdomr.debruin@ucl.ac.uk

**Keywords:** cell cycle, DNA replication stress, CDK, G1/S transcription, checkpoint, genome integrity, Human, *S. cerevisiae*, *S. pombe*

## Abstract

New data on the relationship between two proteins, cyclin D and Rb, suggest that we need to re-evaluate our understanding of how cells enter into the cell cycle.

**Related research article** Narasimha AM, Kaulich M, Shapiro GS, Choi YJ, Sicinski P, Dowdy SF. 2014. Cyclin D activates the Rb tumor suppressor by mono-phosphorylation. *eLife* **3**:e02872. doi: 10.7554/eLife.02872

**Image** How do cells decide when to divide?

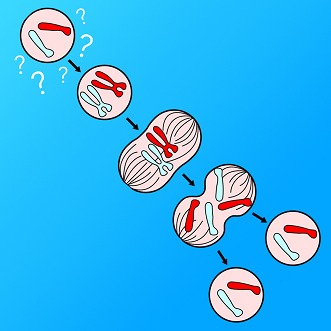

Cell division is a process that is essential to the propagation of life and involves many different steps. In plants and animals this cell cycle begins with the cell growing and producing proteins. Then the cell duplicates its DNA, and equally divides its genetic material between the two daughter cells, before these cells finally separate from each other. The transitions in the cell cycle are tightly controlled to guarantee that the right events take place in the right order (Figure 1A).

**Figure 1.**
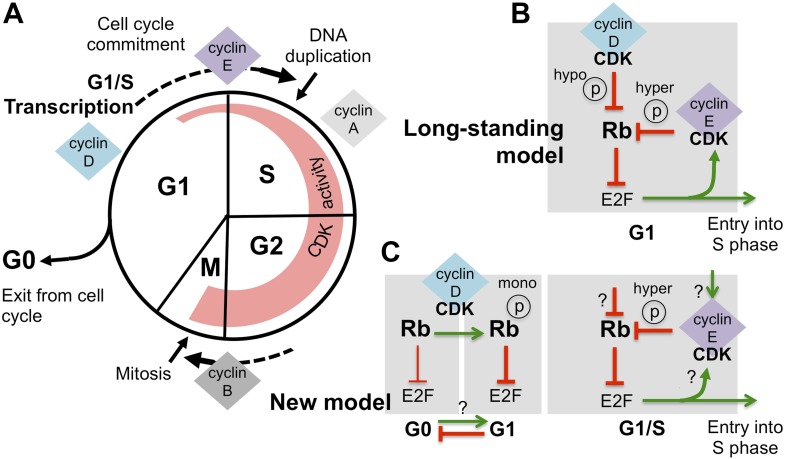
Revising the role of cyclin D/CDK in the regulation of Rb activity. (**A**) The cell cycle involves the following phases: the S-phase, during which DNA is duplicated, and the M-phase (or mitosis), during which chromosome segregation and cell division happen. A gap or G1-phase precedes the S-phase, and the G2-phase happens before the M-phase. Cyclins and cyclin-dependent kinases (CDKs) form a complex that drives progress through the cell cycle: the activity of these complexes (indicated by the width of the red region) starts in G1-phase and increases throughout the cell cycle, until the cyclins are destroyed during the M-phase. Cyclin-CDK complexes drive the G1-to-S transition (cell cycle commitment) by activating the E2F transcription factors and triggering large-scale changes in gene expression. (**B**) The long-standing model of how E2F-transcription is activated is as follows: initial hypo-phosphorylation (hypo ℗) and gradual inactivation of Rb by cyclin D/CDK leads to an accumulation of cyclin E. This triggers a positive feedback loop, as more cyclin E/CDK results in hyper-phosphorylation (hyper ℗) and complete inactivation of Rb, which activates E2F-dependent transcription. (**C**) The work by Dowdy and co-workers establishes a new role for cyclin D/CDK in the regulation of Rb activity by demonstrating that mono-phosphorylation (mono ℗) of Rb by cyclin D/CDK in fact activates Rb to repress E2F transcription. Mono-phosphorylated Rb is the active form in dividing cells, and it is throught that un-phosphorylated Rb is involved in exiting from the cell cycle into a resting or ‘G0-phase’ (the thickness of the inhibition arrows reflects strength of Rb activity). Based on these results Dowdy and co-workers suggest that an increase in cyclin D/CDK activity allows cells to enter the G1-phase and be primed for cell cycle progression. Whether mono-phosphorylation prevents exit from the cell cycle or promotes entry into G1 phase is currently unknown (indicated by ‘?’). Hyper-phosphorylation by cyclin E/CDK inactivates Rb and activates E2F transcription, without the involvement of cyclin D/CDK. However, the mechanism responsible for triggering the accumulation of cyclin E/CDK is unknown (possible ways are indicated by ‘?’).

Our understanding of how progress through the cell cycle is controlled was greatly improved by the discovery of proteins called cyclins and enzymes called cyclin-dependent kinases (or CDKs for short). During the different stages of the cycle, various cyclins and CDKs form complexes that add phosphate groups to other proteins to drive the cell through the cell cycle (Morgan, 2007).

A cell essentially ‘decides’ to divide when it is in G1-phase; and when it commits to entering the cell cycle, the cell enters S-phase and starts duplicating its DNA. Cues from inside the cell and from the surrounding tissue are involved in this decision, with a tumor suppressor protein called Retinoblastoma protein (or Rb for short) having a central role. It was discovered, more than 20 years ago, that Rb inhibits the activity of a set of transcription factors called E2F that switch genes ‘on’ or ‘off’ during G1-phase (reviewed in Dick and Rubin, 2013; Henley and Dick, 2012). This suggested that the decision to enter a new cell cycle coincides with the E2F transcription factors changing the expression of genes in G1-phase. Subsequent studies suggested that, during this phase, the Rb protein is inactivated in a step-wise manner by CDKs as these enzymes become more active. Rb inactivation was shown to be driven by an increase in the levels of CDK activity associated initially with cyclin D, and then with cyclin E during G1-phase (DeCaprio et al., 1989; Mittnacht et al., 1994; Lundberg and Weinberg, 1998).

Now, in *eLife*, Steven Dowdy and co-workers at the University of California San Diego and Harvard Medical School report results that will force us to revise our understanding of the relationship between the cyclin D/CDK complex and the Rb protein (Narasimha et al., 2014).

According to the long-standing model (Figure 1B), as the level of cyclin D/CDK complexes increases during G1-phase, these complexes gradually add phosphate groups to multiple sites on the Rb protein, a process termed hypo-phosphorylation (Mittnacht et al., 1994). Hypo-phosphorylated Rb protein then becomes less able to inhibit the E2F transcription factors, which are then able to switch on many genes, including the gene that encodes cyclin E. The expression of cyclin E further increases the overall activity of CDKs, causing hyper-phosphorylation of the Rb protein, which fully inactivates this protein, and allows the expression of E2F-dependent genes. This positive feedback loop then drives the cell into a new cell cycle (Johnson and Skotheim, 2013).

Dowdy and co-workers—including Anil Narasimha, Manuel Kaulich and Gary Shapiro as joint first authors—provide new data that contradict the dogma; cyclin D/CDK complexes do not inactivate the Rb protein. Instead, surprisingly, the Rb protein is activated in G1-phase by cyclin D/CDK adding a phosphate group at one site (out of a possible 14). The hyper-phosphorylation of Rb by cyclin E/CDK, which inactivates the Rb protein and activates E2F-dependent transcription, is independent from its mono-phosphorylation by cyclin D/CDK (Figure 1C). The results of Dowdy and co-workers radically change the way we think about the role of cyclin D/CDK in regulating the function of the Rb proteins.

First and foremost the new results challenge the current model in which cyclin D/CDK starts the process of E2F-dependent transcription, which causes the accumulation of cyclin E/CDK and triggers the positive feedback mechanism that commits a cell to dividing. So, how is E2F activity initiated? This is now an open question. Interestingly, it was recently shown that in cells that are undergoing continuous cell division, there is residual cyclin/CDK activity from the previous cell cycle that could trigger the next cell cycle (Spencer et al., 2013). Alternatively, other transcription factors could also be involved in starting cyclin E expression (Naetar et al., 2014).

Furthermore, the Rb protein is activated when DNA is damaged, and Dowdy and co-workers showed that this activity also requires mono-phosphorylation of Rb by cyclin D/CDK. Surprisingly, mono-phosphorylation also takes place in cells that have exited the cell cycle when they are exposed to chemicals that damage DNA. This suggests that the ‘DNA damage checkpoint’ (the pathway that halts the cell cycle to give a cell time to repair its DNA when it is damaged) activates cyclin D/CDKs. However, future work is needed to establish how this occurs in a cell. It will also be important to investigate if the other Rb-related proteins also need to be mono-phosphorylated by cyclin D/CDKs to be activated.

Finally, while Dowdy and co-workers show that mono-phosphorylated Rb is the active form of this protein in cells that are repeatedly dividing, they also present data that suggest that un-phosphorylated Rb is involved in exiting the cell cycle when a cell starts to specialise into a specific cell type. These results support a new model in which an increase in cyclin D/CDK activity, which is seen in many cancers, might ‘prime’ a cell for entry into the cell cycle, by preventing its exit.

Every once in a while a study comes along that changes everything. The work of Dowdy and co-workers represents one of those rare cases that turn a long-standing model on its head. Their results will force those studying the process of commitment to cell division to revisit old data and will initiate new investigations, kicking off an exciting rebirth of a well-established field.

## References

[bib1] DeCaprio JA Ludlow JW Lynch D Furukawa Y Griffin J Piwnica-Worms H Huang CM Livingston DM 1989 The product of the retinoblastoma susceptibility gene has properties of a cell cycle regulatory element. Cell 58:1085–1095. doi: 10.1016/0092-8674(89)90507-2 2673542

[bib2] Dick FA Rubin SM 2013 Molecular mechanisms underlying RB protein function. Nature Reviews Molecular Cell Biology 14:297–306. doi: 10.1038/nrm3567 PMC4754300 23594950

[bib3] Henley SA Dick FA 2012 The retinoblastoma family of proteins and their regulatory functions in the mammalian cell division cycle. Cell Division 7:10. doi: 10.1186/1747-1028-7-10 22417103 PMC3325851

[bib4] Johnson A Skotheim JM 2013 Start and the restriction point. Current Opinion in Cell Biology 25:717–723. doi: 10.1016/j.ceb.2013.07.010 23916770 PMC3836907

[bib5] Lundberg AS Weinberg RA 1998 Functional inactivation of the retinoblastoma protein requires sequential modification by at least two distinct cyclin-cdk complexes. Molecular and Cellular Biology 18:753–761 9447971 10.1128/mcb.18.2.753 PMC108786

[bib6] Mittnacht S Lees JA Desai D Harlow E Morgan DO Weinberg RA 1994 Distinct sub-populations of the retinoblastoma protein show a distinct pattern of phosphorylation. EMBO Journal 13:118–127 8306955 10.1002/j.1460-2075.1994.tb06241.x PMC394785

[bib7] Morgan DO 2007 The cell cycle; principles of control. In: Lawrence E, editor. New Science Press Ltd

[bib8] Naetar N Soundarapandian V Litovchick L Goguen KL Sablina AA Bowman-Colin C Sicinski P Hahn WC DeCaprio JA Livingston DM 2014 PP2A-mediated regulation of Ras signaling in G2 is essential for stable quiescence and normal G1 length. Molecular Cell 54:932–945. doi: 10.1016/j.molcel.2014.04.023 24857551 PMC4118046

[bib9] Narasimha AM Kaulich M Shapiro GS Choi YJ Sicinski P Dowdy SF 2014 Cyclin D activates the Rb tumor suppressor by mono-phosphorylation. eLife 3:e02872. doi: 10.7554/eLife.02872 24876129 PMC4076869

[bib10] Spencer SL Cappell SD Tsai FC Overton KW Wang CL Meyer T 2013 The proliferation-quiescence decision is controlled by a bifurcation in CDK2 activity at mitotic exit. Cell 155:369–383. doi: 10.1016/j.cell.2013.08.062 24075009 PMC4001917

